# Comparative antiseizure medications of adjunctive treatment for children with drug-resistant focal-onset seizures: A systematic review and network meta-analysis

**DOI:** 10.3389/fphar.2022.978876

**Published:** 2022-12-16

**Authors:** Lanlan Zhang, Yuehong Li, Weikai Wang, Chengzhong Wang

**Affiliations:** Department of Pediatrics, Yancheng Maternal and Children’s Health Hospital, Yancheng, China

**Keywords:** focal onset seizure, network meta-analysis, anti-seizure medication, epilepsy, children

## Abstract

**Purpose:** In this study, we intended to compare and rank the efficacy and acceptability of antiseizure medications (ASMs) for adjunctive treatment of children with drug-resistant focal-onset seizures.

**Method:** We conducted a computerized search of PubMed, EMBASE, Cochrane Library, Web of Science, and Google Scholar to identify eligible randomized controlled trials (RCTs) published before 31 May 2022. We included studies evaluating the efficacy and tolerability of antiseizure medications for children with drug-resistant focal-onset seizures. The efficacy and safety were reported in terms of responder and dropout rate along with serious adverse events, the outcomes were ranked with the surface under the cumulative ranking curve (SUCRA).

**Results:** A total of 14 studies (16 trials) with 2,464 patients were included, involving 10 active antiseizure medications. For the primary endpoint of at least 50% reduction in focal-onset seizures, the surface under the cumulative ranking curve ranking suggested that lamotrigine and levetiracetam were more effective as compared with other antiseizure medications; moreover, levetiracetam had the highest probability of rank first for achieving seizure freedom. Concerning tolerability, oxcarbazepine and eslicarbazepine acetate were associated with higher dropout rates relative to other antiseizure medications and placebo, and topiramate was associated with higher occurrence of side effects. No significant differences were found between active antiseizure medications concerning dropout for side effects.

**Conclusion:** According to the surface under the cumulative ranking curve ranking, lamotrigine, levetiracetam, and oxcarbazepine were more efficacious than other active antiseizure medications in terms of responder rate. Concerning tolerability, oxcarbazepine was more likely to lead to dropout and topiramate was associated with higher occurrence of side effects.

## Introduction

Epilepsy is a common chronic neurological disorder characterized by recurrent, unprovoked seizures, and the goal for the management of this disorder is primarily to reduce the onset frequency (Fisher et al., 2017). Epilepsy affects individuals of any age and ethnicity and has deleterious effects on social, vocational, physical, and psychological wellbeing (Bell et al., 2014). It is estimated that in 3.5 million people develop epilepsy worldwide every year, 40% of whom are children younger than 15 years (Guerrini, 2006). Among the varied epilepsy types in children and adolescents, focal-onset seizures are the most frequent forms and can be caused by various acquired or genetic etiologies (Wilmshurst et al., 2014).

Although most epilepsy will reach remission after treatment with mono or adjunctive therapy of ASMs, attempts at drug withdrawal after 3 years of seizure control are followed by relapse in as many as 25% of patients (Schmidt and Gram, 1996). Some studies showed that the second-generation ASMs are more efficacious than the first-generation AEDs and appear better tolerability; however, 25%–30% of children with epilepsy are still refractory to these wider treatment options (Kwan et al., 2010; Verrotti et al., 2011). Therefore, older ASMs continue to play a major role in the current treatment of epilepsy. The ASM selection strategy needs to consider various factors such as age, gender, comorbidities, side effects, pharmacokinetics, and drug-drug interaction (Arzimanoglou et al., 2018). The goal for the treatment of epilepsy is to achieve seizure freedom, minimal or no drug-related side effects, and the best quality of life. Nevertheless, for drug-resistant seizures, instead of achieving complete control the goal maybe substantial seizure onset reduction. In pediatric patients, major concerns include cognitive, neuropsychological, psychiatric, and behavioral side effects (Leviton et al., 2013). In recent years, several RCTs of ASM for the treatment of pediatric focal-onset seizures provided clinicians with new options (Arzimanoglou et al., 2018). Nevertheless, in the absence of head-to-head comparison trials, these various choices make it difficult to choose optimal treatments. Although several previous network meta-analyses assessed the efficacy and tolerability of ASMs have been published, they did not differentiate children from adults or did not include newer ASMs (Rosati et al., 2017; Hu et al., 2018). Thus, in the current network meta-analysis we aimed to systematically compare and rate the efficacy and acceptability of currently available ASM for pediatric drug-resistant focal-onset seizures.

## Materials and methods

This study was performed using predefined literature screening and data extraction protocols, and reported according to the Preferred Reporting Items for Systematic Reviews and Meta-Analyses (PRISMA) extension statement for network meta-analyses (Hutton et al., 2015). The primary outcome was the proportion of patients achieving at least 50% reduction (responder rate) of seizure onset frequency during the overall treatment period (titration and maintenance phase) (Birbeck et al., 2002; Kwan et al., 2011). Secondary outcomes include seizure freedom rate, acceptability profile in terms of dropout for any reason and side effects along with the serious adverse events induced by treatment drugs.

### Search strategy and selection criteria

A literature search of PubMed, EMBASE, Cochrane Library, Web of Science, and Google Scholar online scientific publication databases was performed up to 31 May 2022. We included RCT for assessing ASMs for the treatment of drug-resistant focal-onset seizures in children, with no language restriction. The terms combined synonyms used for searching were as follows: [(child*) OR (adolescent*) OR (pediatric)] AND [(focal*) OR (partial*)] AND [(*onset) OR (seizure*) OR (epilepsy)]. An additional search was performed by manually screening the bibliographies of the reviews and included articles. Besides, we retrieved trials reporting results from the US National Institutes of Health database of clinical trials and the World Health Organization International Controlled Trials Registry website up to 31 May 2022. Studies identified by the literature search were assessed by two independent reviewers, and disagreements were resolved by consensus *via* discussion with a third reviewer.

Studies that met all of the following criteria would be included: 1) full-length article reporting the randomized double-blinded controlled trial for the treatment of children with drug-resistant focal-onset seizures, 2) participants were diagnosed with drug-resistant focal-onset seizures based on clinicians’ opinions, 3) for evaluating the efficacy of any dose of the drugs of interest, and compared the ASM to placebo or with each other, 4) providing sufficient details to evaluate the efficacy and tolerability. Studies that satisfied any of the following criteria would be excluded: 1) retrospective or observational studies, 2) for treatment of other epilepsy forms rather than focal-onset seizures, 3) case reports and case series, 4) did not provide details allowing for assessment of efficacy and acceptability, 5) comments, editorials, reviews, meeting abstracts, letters, and guidelines.

### Data extraction and quality assessment

Two reviewers independently extracted the following information from RCTs with a standardized protocol: authors, publication year, location of trials conducted, each treatment group sample size, mean (or median) age, male/female ratio, follow-up period, details regarding efficacy such as responder and seizure freedom rate, as well as tolerability such as dropouts for any reason and side effects, and the incidence of serious adverse events related to treatments. We assessed the quality of included trials using the Cochrane Collaboration’s tool, with which each RCT was assigned as high, low, or unclear risk of bias according to the following criteria: random sequence generation, allocation concealment, blinding of outcome participants and personnel, blinding of outcome assessment, incomplete outcome data, selective reporting, and other bias (Higgins et al., 2011). The quality assessment was performed by two reviewers (*Z.L.L.* and *L.Y.H.*) and disagreements were resolved by discussion and consultation with the third reviewer (*W.C.Z.*).

### Data synthesis and analysis

We used the frequentist model to perform network meta-analysis in all RCTs of interest. For graphically demonstrating the comparison between ASMs, the network plot was constructed in which the node sizes represented study participants and connection widths correspond to the number of studies reporting the corresponding comparison. To compare the efficacy and acceptability between ASMs, we estimated summary odds ratios (ORs) and corresponding 95% confidence interval (95% CI) with a random-effects network meta-analysis model. The inconsistency assumption was used to determine the level of disagreement between direct and indirect evidence, which was evaluated using the overall inconsistency test by fitting design-by-treatment in the inconsistency model (Dias et al., 2010). In the case of different intervention doses in a single RCT, we combined these doses into a single intervention group. The SUCRA and the mean ranks were used to rate the treatments, which represented the probability of a given treatment being the best (or worst) option (Salanti et al., 2011). For SUCRA values, 0% indicates no chance the treatment is the most efficacious, and 100% indicates the treatment is certainly the most efficacious. As no concrete methodology to evaluate the publication bias between studies in network meta-analysis, we used the comparison-adjusted funnel plots to assess the publication bias among treatment comparisons (Chaimani and Salanti, 2012). Comparison-adjusted funnel plots are scatter plots of effect size versus precision, in which the substantial asymmetry around the effect estimate suggests the likelihood of publication bias. Heterogeneity across included RCTs was determined with the Cochran’s *Q* test and measured with *I* (Bell et al., 2014) statistic: 0%–40%, slight; 30%–60%, moderate; 50%–90%, substantial; and 75%–100%, considerable (Higgins et al., 2003). All analyses were conducted using STATA, version 15.1 (StataCorp, United States), with *p* < .05 indicated statistically significant.

## Result

### Literature search

**FIGURE 1:**
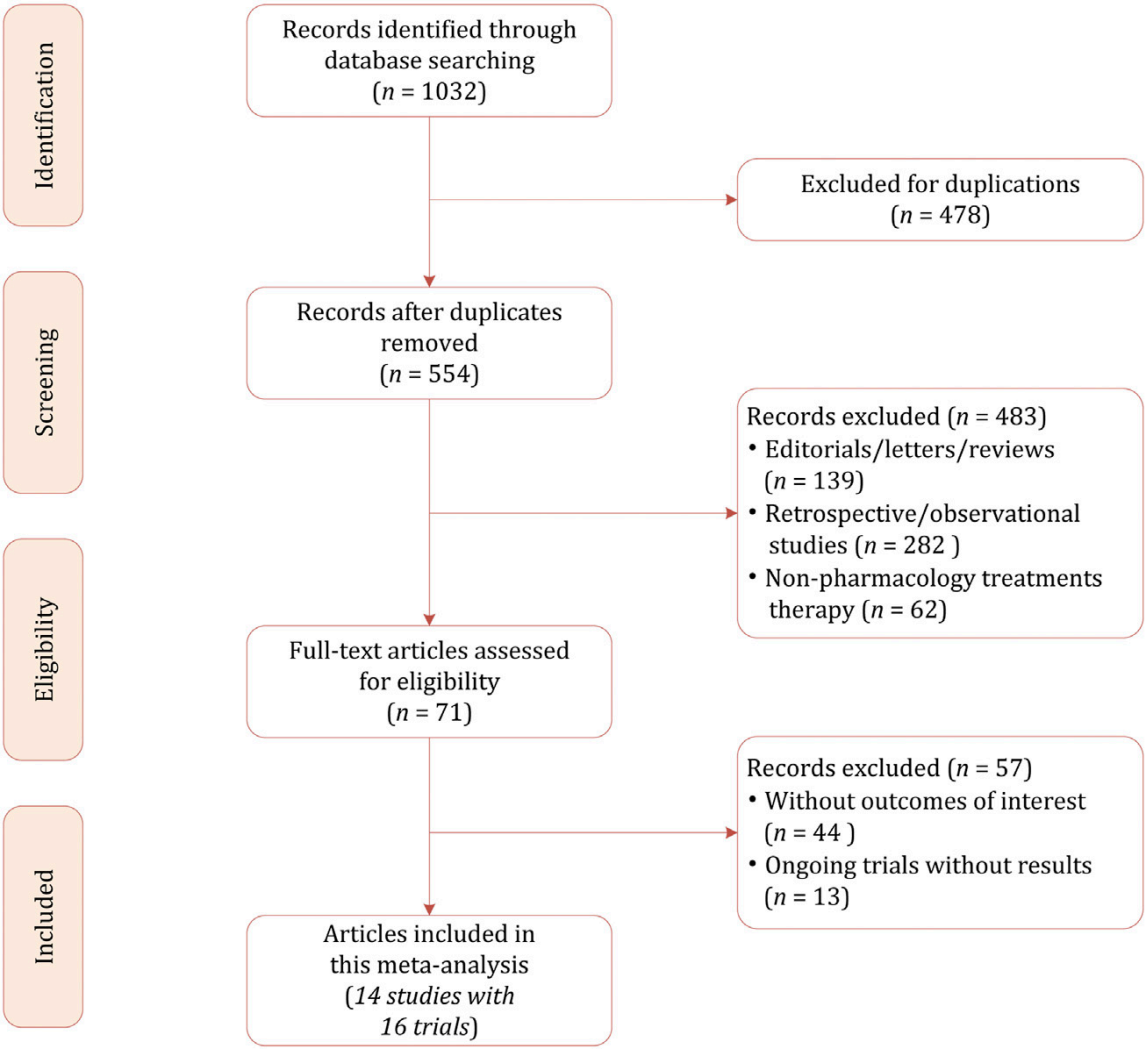
Literature selection process.

Our initial literature search identified a total of 1,032 citations, of which 478 were excluded because of duplicates. After reviewing the titles and abstracts, 483 studies were excluded for not in the field of interest, non-pharmacology treatment, retrospective and observational in study design. The full-text review was conducted in the potentially eligible 71 studies, finally 14 studies with a total of 2,464 participants were included in this network meta-analysis (Lagae et al., 2016; Glauser et al., 2006; Glauser et al., 2000; Duchowny et al., 1999; Elterman et al., 1999; Piña-Garza et al., 2009; Farkas et al., 2019; Appleton et al., 1999; Novotny et al., 2010; Levisohn et al., 2009; Rosenfeld et al., 2015; NCT01527513. Effects of Eslicarbazepine Acetate, 1527; NCT00988156. Eslicarbazepine Acetate; NCT00975715. Efficacy). The PRISMA flowchart demonstrating the study selection process is presented in Figure 1.

### Study characteristics and quality assessment

Table 1 summarizes the demographic characteristics of included studies. The study sample ranged from 86 to 343, with an age of 1 month–18 years. In total 1,352 participants were randomly assigned to the active ASMs, and 1,112 participants were randomly assigned to placebo. In the majority of studies the treatment period ranged from 12 to 19 weeks, whereas in one RCT that involved infants younger than 4 years the treatment period merely lasted for 3 weeks (Piña-Garza et al., 2009). Concerning specific ASMs, perampanel was reported in four trials, levetiracetam was reported in three trials, oxcarbazepine, topiramate, perampanel, and eslicarbazepine acetate were reported in each 2 trials, and the remaining three ASMs was reported by each 1 trial. Regarding the baseline period, in most trials it lasted for 4–8 weeks, whereas in two trials this phase merely lasted for 48 h (Piña-Garza et al., 2009; Novotny et al., 2010). In the majority of patients the number of concomitant ASMs ranged from 1 to 3.

**TABLE 1:** Characteristics of included studies.

Study	ASM	Year	Number (active/placebo)	Age (year, mean ± SD)	Gender (M/F)	Baseline seizure frequency (28 days, mean/median)	Concomitant ASMs	Dosage (per day) period (weeks)	Baseline	Follow-up (weeks)
Appleton	Gabapentin	1999	119/128	8.4 ± 2.6	134/113	24.1 (2.7–2,893) 28.0 (1.3–698)	NA	600–1800 mg	4	12
Duchowny	Lamotrigine	1999	98/101	2–16	103/96	NA	NA	1–15 mg/kg	8	18
Elterman	Topiramate	1999	41/45	8.8 ± 3.6/9.0 ± 3.4	48/38	2–232 2–1,133	CBZ/VPA/PHT/GBP/LTG	25–400 mg	8	16
Farkas	Lacosamide	2019	171/172	10.7 (3.5)	190/153	NA	VPA/LEV/CBZ/LTG/TPM/OXZ	100–400 mg	8	16
Glauser	Oxcarbazepine	2000	138/129	11 (3–17)	141/126	12 (3–1,470) 13 (2–554)	CBZ/VPA/LTG/PHT	30–46 mg/kg	8	16
Glauser	Levetiracetam	2006	101/97	10.4 (4–17)/9.7 (3–17)	100/98	4.7 (0–696) 5.3 (0–467)	CBZ/TPM/VPA/LTG	20–60 mg/kg	8	14
Lagae	Perampanel	2016	85/48	14.0 (12–17)	80/53	NA	VPA/LEV/LTG/OXZ	8–12 mg	1	19
Levisohn	Levetiracetam	2009	64/34	4–16	56/42	63 ± 98.4 34 ± 100	OXZ/LTG/VPA/CBZ/TPM/ZNM/PHT/GBP/CLA	20–60 mg/kg	4	12
NCT00975715	Oxcarbazepine	2014	48/51	9.5 ± 2.87	53/46	65.6 ± 109.5 90.6 ± 283.2	NA	NA	4	8
NCT01527513	Eslicarbazepine Acetate	2014	83/40	6–16	73/50	5.0 (2–140) 4.5 (2–345)	VPA/CBZ/LTG/LEV/TPM	10–30 mg/kg (max 1,200 mg)	4	12
NCT00988156	Eslicarbazepine Acetate	2018	134/129	2–18	126/137	11.5 (3.7–605.8) 17.0 (3.9–1972.5)	VPA/CBZ/LTG/LEV/TPM	10–30 mg/kg (max 1,200 mg)	8	18
Novotny	Topiramate	2010	112/37	11.7/12	78/71	7.5 (0.0–175.0) 5.0 (0.0–78.5) 8.0 (0.0–100.0) 6.0 (0.0–240.0)	VPA/PHE/CBZ	5–25 mg/kg	48 h	3
Piña-Garza	Levetiracetam	2009	60/56	1–48 m	57/59	15.2 (4.5–39.0)* 6.8 (2.0–16.2)	VPA/PHE/TPM/OXZ/VGB/CLZ/CBZ/CNZ	20–50 mg/kg	48 h	3
Rosenfeld	Perampanel	2015	98/45	12–17	84/59	NA	CBZ/VPA/LTG/LEV	2–12 mg	6	19

**Abbreviations**: ASMs, anti-seizure medications; CBZ, carbamazepine; CLZ, clobazam; CLA, clorazepic acid; CNZ, clonazepam; GBP, gabapentin; LEV, levetiracetam; LTG, lamotrigine; OXZ, oxcarbazepine; PHE, phenobarbital; PHT, phenytoin; RFM, rufinamide; TPM, topiramate; VGB, vigabatrin; VPA, valproate; ZNM, zonisamide; SD, standard deviation. Notes: * The baseline seizure frequency are 24–48 h.

The overall quality assessment was not very high. Of the 16 trials included in the network meta-analysis, only half were assigned at low risk of bias. Of the remaining eight trials, one was rated as unclear risk of bias because of too short baseline and treatment period (Piña-Garza et al., 2009). In addition, three trials were from clinical trials and the details were not reported, thus were rated as unclear risk of bias. (NCT00988156. Eslicarbazepine Acetate; NCT00975715. Efficacy; Chen et al., 2020); Supplementary Figure S1 demonstrated the detailed risk of bias for included RCTs. The results of the comparison-adjusted funnel plots showed that there was no evidence of apparent asymmetry, indicating no significant publication bias (Supplementary Figure S2).

### Comparison of efficacy and acceptability

**FIGURE 2:**
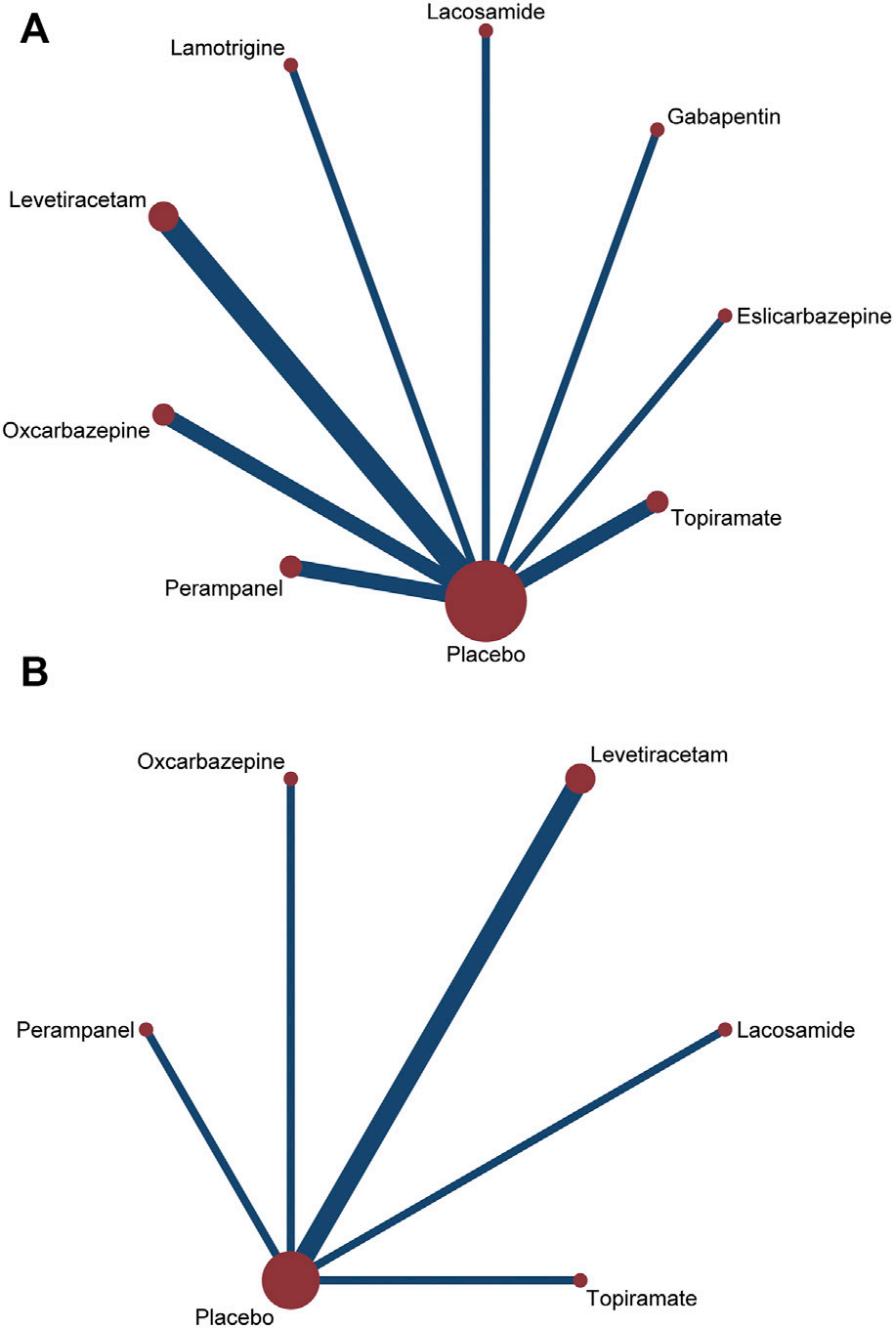
Network plots of treatment comparisons for the efficacy outcomes. Circle size is proportional to the number of study participants assigned to receive each intervention. The line width corresponds to the number of studies comparing the treatments. **(A)** ≥50% reduction of seizure frequencey; **(B)** seizure freedom.

**FIGURE 3:**
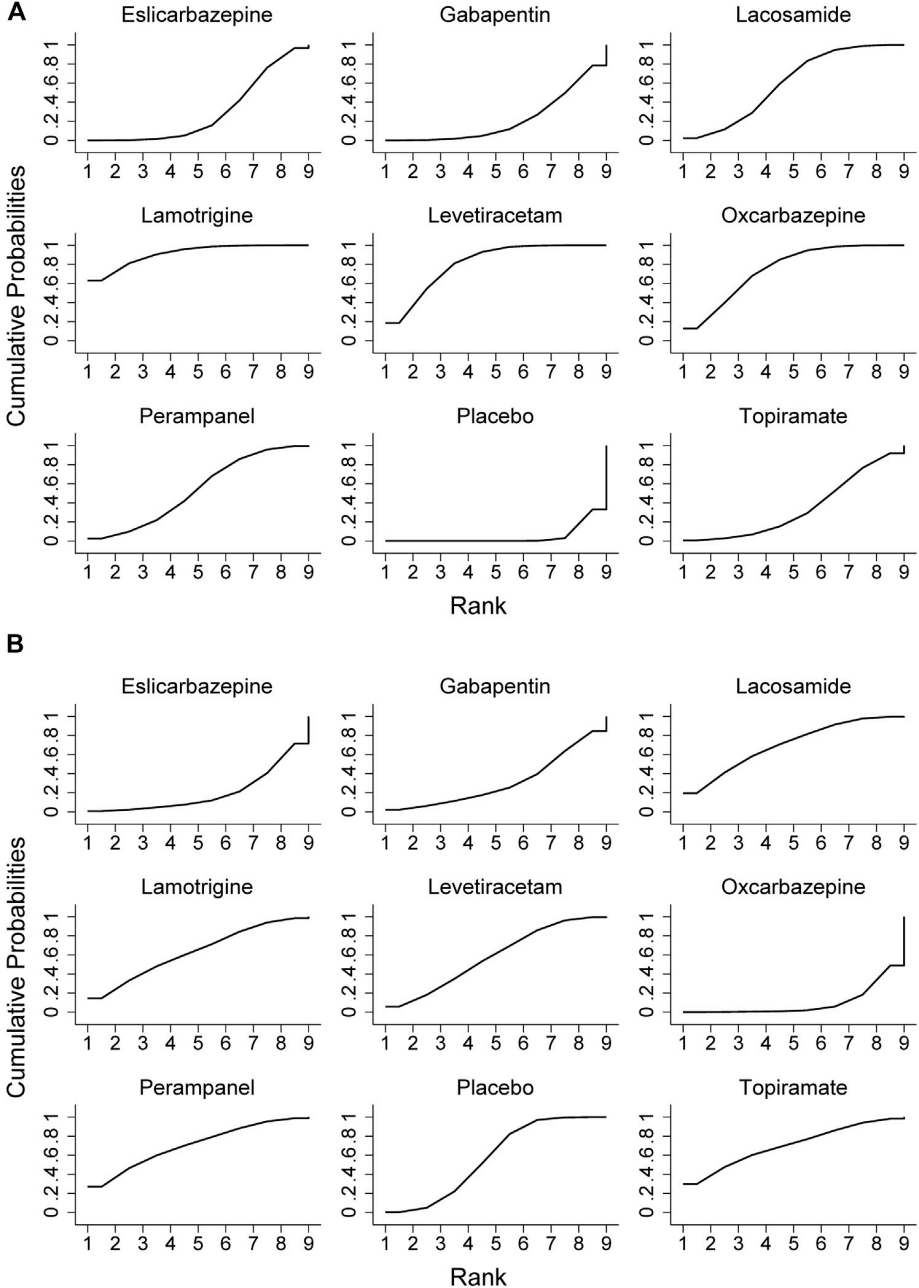
Surface under the cumulative ranking curve probabilities for the ranking. **(A)** ≥50% reduction of seizure frequencey; **(B)** dropout rate for side effects.

All trials reported the primary outcome of patients achieving 50% seizure onset reduction, and the corresponding network plot is presented in Figure 2A. Table 2 shows the results of the network meta-analysis, because all trials were comparisons between antiseizure medications and placebo, it was unfeasible to check for inconsistency between direct and indirect treatments. There was no statistical heterogeneity between studies (I^2^ = 0.0%). Except for topiramate, eslicarbazepine acetate, and gabapentin, all other ASMs were significantly more efficacious than placebo. According to the SUCRA lamotrigine (91.1%) had the greatest likelihood rank first, and following were levetiracetam (80.6%) and oxcarbazepine (74.8%), the corresponding SUCRA ranking are present in Figure 3A. A total of seven trials reported the endpoint of patients achieving complete seizure freedom, the network plot is demonstrated in Figure 2B. I^2^ = 0.0% showed no statistical heterogeneity when data were pooled. Among the five ASMs, only levetiracetam (OR 5.62, 95% CI 2.39–13.22, *p* < .001) was significantly superior to placebo, and the SUCRA ranking suggested that levetiracetam (80.4%) had the highest probability rank first among these active ASMs (Supplementary Figure S3).

**TABLE 2:** Network analysis of efficacy (responder rate).

Lamotrigine	0.78 (0.33, 1.82)	0.74 (0.29,1 .86)	0.59 (0.23, 1.53)	0.59 (0.24, 1.41)	0.53 (0.24, 1.18)	0.41 (0.15, 1.08)	0.39 (0.16, 0.92)	0.35 (0.15,0.84)	0.34 (0.12, 0.90)	0.26 (0.13, 0.53)
1.29 (0.55, 3.02)	Levetiracetam	0.95 (0.44, 2.03)	0.76 (0.35, 1.66)	0.76 (0.38, 1.50)	0.68 (0.37, 1.24)	0.52 (0.23, 1.19)	0.50 (0.25, 0.98)	0.46 (0.23, 0.89)	0.43 (0.19, 0.99)	0.34 (0.21, 0.54)
1.36 (0.54, 3.44)	1.06 (0.49, 2.26)	Oxcarbazepine	0.81 (0.34, 1.91)	0.80 (0.37, 1.74)	0.72 (0.37, 1.38)	0.55 (0.23, 1.34)	0.52 (0.24, 1.14)	0.48 (0.22, 1.06)	0.46 (0.18, 1.13)	0.36 (0.20, 0.64)
1.69 (0.65, 4.35)	1.31 (0.60, 2.85)	1.24 (0.52, 2.93)	Zonisamide	0.99 (0.44, 2.20)	0.89 (0.43, 1.83)	0.68 (0.27, 1.71)	0.65 (0.29, 1.44)	0.60 (0.27, 1.32)	0.57 (0.22, 1.43)	0.44 (0.24, 0.82)
1.70 (0.71, 4.09)	1.32 (0.67, 2.63)	1.25 (0.58, 2.73)	1.01 (0.45, 2.25)	Lacosamide	0.90 (0.48, 1.68)	0.69 (0.30, 1.60)	0.66 (0.32, 1.34)	0.60 (0.30, 1.22)	0.57 (0.24, 1.34)	0.45 (0.27, 0.74)
1.89 (0.85, 4.22)	1.47 (0.81, 2.67)	1.39 (0.72, 2.68)	1.12 (0.55, 2.31)	1.11 (0.60, 2.07)	Perampanel	0.77 (0.36, 1.64)	0.73 (0.39, 1.35)	0.67 (0.36, 1.26)	0.63 (0.29, 1.38)	0.50 (0.34, 0.71)
2.46 (0.92, 6.56)	1.91 (0.84, 4.33)	1.81 (0.74, 4.40)	1.46 (0.58, 3.65)	1.44 (0.62, 3.34)	1.30 (0.61, 2.77)	Topiramate	0.95 (0.41, 2.19)	0.87 (0.38,2.01)	0.83 (0.32, 2.15)	0.64 (0.33, 1.26)
**2.59 (1.09, 6.19)**	**2.01 (1.02, 3.98)**	1.91 (0.88, 4.14)	1.54 (0.69, 3.41)	1.52 (0.75, 3.09)	1.37 (0.74, 2.54)	1.05 (0.46, 2.43)	Eslicarbazepine	0.92 (0.46, 1.84)	0.87 (0.37, 2.03)	0.68 (0.41, 1.12)
**2.82 (1.19, 6.69)**	**2.19 (1.12, 4.28)**	2.07 (0.94, 4.58)	1.67 (0.76, 3.69)	1.65 (0.82, 3.34)	1.49 (0.79, 2.80)	1.15 (0.50, 2.64)	1.09 (0.54, 2.18)	Pregabalin	0.95 (0.41, 2.19)	0.74 (0.45, 1.20)
**2.98 (1.11, 8.02)**	**2.32 (1.01, 5.30)**	2.19 (0.89, 5.43)	1.77 (0.70, 4.46)	1.75 (0.75, 4.10)	1.58 (0.73, 3.42)	1.21 (0.46, 3.16)	1.15 (0.49, 2.68)	1.06 (0.46, 2.45)	Gabapentin	0.78 (0.39, 1.55)
**3.82 (1.87, 7.81)**	**2.97 (1.86, 4.73)**	**2.81 (1.55, 5.09)**	**2.27 (1.22, 4.22)**	**2.24 (1.35, 3.71)**	**2.02 (1.40, 2.90)**	1.55 (0.79, 3.04)	1.47 (0.90, 2.43)	1.36 (0.83, 2.20)	1.28 (0.65, 2.54)	Placebo

Network meta-analysis results of the efficacy in terms of odd ratio (OR) for responder rate, which are reported in order of surface under the curve cumulative ranking. Top-ranked treatment listed in the top left corner and rankings proceed down the diagonal. Bold values indicate statistically significant results.

**FIGURE 4:**
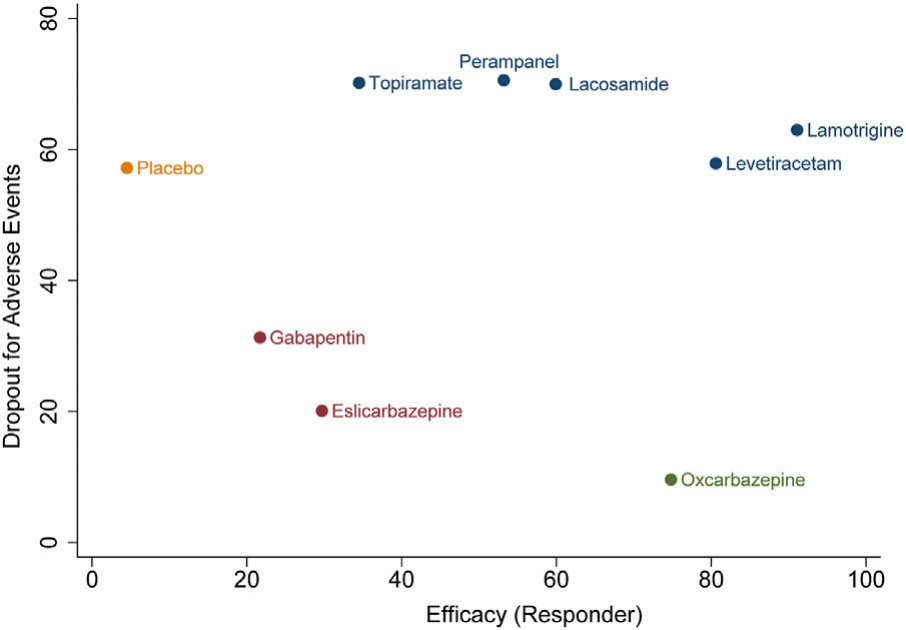
Clustered ranking plot of the antiseizure medications network for the efficacy and tolerability outcomes. A, seizure responder and any occurrence of at least one treatment-emergent adverse event leading to treatment discontinuation. Ranking plots are based on cluster analysis of SUCRA values for two different outcomes: efficacy and tolerability.

In terms of acceptability, all trials reported that the number of patients experienced adverse effects. We did not observe statistical heterogeneity across trials (I^2^ = 0.0%). Our analyses revealed that significantly higher proportion of patients allocated to topiramate (OR 4.11, 95% CI 1.74–9.7, *p* = .001), oxcarbazepine (OR 2.42, 95% CI 1.32–4.43, *p* = .004), and gabapentin (OR 1.99, 95% CI 1.03–3.82, *p* = .04) experienced side effects as compared to placebo. The SUCRA ranking suggested that lamotrigine (87.9%) has lower occurrence of side effect than other ASMs (Supplementary Table S1). Regarding the endpoint of dropouts for any reason, no significant difference was found between active ASMs and placebo, and no statistical heterogeneity (I^2^ = 0.0%) was observed among trials. Non-etheless, patients treated with oxcarbazepine lead to significantly higher dropout rate relative to topiramate (OR 9.06, 95% CI 1.43–57.38, *p* = .02) and perampanel (OR 5.84, 95% CI 1.12–30.53, *p* = .04). Regarding the dropout due to side effects, oxcarbazepine had a significantly higher dropout rate relative to placebo (OR 4.77, 95% CI 1.32–17.28, *p* = .017) and lacosamide (OR 6.9, 95% CI 1.05–45.29, *p* = .04) (Figure 3B). We did not observe statistical heterogeneity (I^2^ = 28.4%) throughout the trials. The incidence of serious adverse events (SAE) was reported in eight ASMs from 18 trials, and no significant difference between ASMs and placebo. The SUCRA ranking revealed that topiramate (74.7%), lacosamide (67.5%), and perampanel (67.0%) were less likely than other ASMs to lead to an SAE. The results of the network meta-analysis for dropout for any reason and side effects are presented in Supplementary Tables S2, S3. Due to insufficient data, it was unfeasible to perform the comparison between ASMs or placebo regarding specific side effects; however, we presented them in Supplementary Table S4. The clustered ranking plots of the ASM network for the efficacy (responder rate) and tolerability (dropout for side effects) for individual ASMs are shown in Figure 4.

## Discussion

To our knowledge, this network meta-analysis represents the most comprehensive comparative synthesis to date regarding the efficacy and tolerability of adjunctive ASMs for children with drug-resistant focal-onset seizures. We have addressed the limitations of previous network meta-analyses that did not differentiate children from adults or not included newer ASMs (Rosati et al., 2017; Hu et al., 2018). On the base of 16 trials, our analysis showed that except for topiramate, eslicarbazepine acetate, and gabapentin, all other ASMs were significantly more efficacious than placebo in terms of responder rate. The SUCRA ranking revealed that lamotrigine and levetiracetam had a relatively higher responder as compared with other active ASMs. Moreover, these two ASMs were significantly superior to eslicarbazepine acetate and gabapentin. With seizure freedom as the endpoint, levetiracetam had the highest probability rank first and was the only ASM significantly superior to placebo, demonstrating high efficacy for the treatment of focal-onset seizures. Concerning tolerability, oxcarbazepine and eslicarbazepine acetate were associated with higher dropout rate relative to other ASMs and placebo, and topiramate was associated with higher occurrence of side effects. Data for the individual adverse events were scarce, which precluded further analysis of this endpoint in the current study. The rational management of newly diagnosed epilepsy is based on the prescription of a single ASM. Use of monotherapy minimizes the risk of toxicity, facilitates assessment of drug response, and prevents drug interactions. However, among more than 20 second-generation and third-generation ASMs, only a few are approved for monotherapy of pediatric patients with focal-onset seizures (Chen et al., 2020). In one open-label study that compared alternative monotherapy with adjunctive therapy in drug-resistant focal-onset seizures, there was no significant difference between the two groups in terms of achieving seizure freedom after 12 months of treatment (Beghi et al., 2003).

Several previous network meta-analyses have compared ASMs for the treatment of focal-onset epilepsy. However, most of them were focused on adults or with a mixed population, and the only study investigating ASMs for the treatment of focal epilepsy in children merely included six trials (Campos et al., 2016; Rosati et al., 2017; Hu et al., 2018; Lattanzi et al., 2022). In contrast to adulthood, studies demonstrated that children have different pharmacokinetic and pharmacodynamic profiles. Moreover, clinical trials are prone to recruit more severe epilepsy in children than in adults (Crawley et al., 2003; Caldwell et al., 2004). In a previous study, Rheims et al. found that placebo has a greater response in children than in adults, especially for gabapentin, levetiracetam, oxcarbazepine, and topiramate (Rheims et al., 2008). In another study, Hu et al. compared 17 active ASMs for mixed a population of adults and children; however, the primary outcome they used was seizure freedom rather than responder rate, therefore it was unfeasible to compare our results with them (Hu et al., 2018). In a recent study, Lattanzi et al. investigated the efficacy of five third-generation ASMs for the treatment of focal-onset seizures in the adult population, which has similar ranking order with our findings in three ASMs (eslicarbazepine acetate, lacosamide, and perampanel) (Lattanzi et al., 2022). In recent years, cannabidiol and fenfluramine have shown favorable efficacy for the treatment of Dravet syndrome and/or Lennox-Gastaut syndrome demonstrating clinically meaningful reduction in seizure frequency (Lattanzi et al., 2018; Lattanzi et al., 2020; Zhang et al., 2021). However, clarification of the independent effects of cannabidiol therapy and a clobazam comedication effect needs to be addressed. Furthermore, whether cannabidiol has a positive effect on drug-resistant focal seizures remain need high quality RCTs to validate.

As results of this network meta-analysis were derived from indirect comparisons of ASMs between trials, which were not methodologically rigorous. The inclusion criteria may vary among trials especially between older trials and newer ones, hence the conclusion of this network meta-analysis should be interpreted with caution. Nevertheless, in the absence of head-to-head comparison trials, network meta-analysis can provide more precise estimate of the relative efficacy and tolerability than pairwise comparison, and allow treatments to be ranked to assist clinical decisions (Salanti et al., 2008; Salanti, 2012). Our findings represent the best currently available evidence for patients and clinicians to inform first-line and second-line treatment decisions. In summary, our network meta-analysis revealed that lamotrigine, levetiracetam, and oxcarbazepine were more efficacious than other ASMs for the treatment of focal-onset seizures while using responder rate as the endpoint. Because the ranking of efficacy and tolerability were based on mean SUCRA values, it was not representing that higher-ranked ASMs were notably superior to lower-ranked ones. Despite the similarity of the included trials, some differences in characteristics remain existed such as duration of the treatment period and concomitant medications. Generally, trials with shorter treatment periods may have underestimated the occurrence of side effects as compared to those with longer time. Moreover, a higher burden of concomitant ASMs may lead to an increased frequency of side effects.

There are some limitations to our study which should be taken into consideration. First, all trials included were adjunctive treatment, the mechanism of action of concomitant ASMs precludes investigating the potential drug-drug interactions and combinations. However, due to scarce high-quality trials for monotherapy of focal-onset seizures in children, it is unfeasible to compare the efficacy of ASMs with only a few monotherapy trials. Second, for trials conducted 20 years ago or from ClinicalTrials.gov, the inclusion criteria for participants and endpoints used to judge treatment efficacy have not been explicitly described. Therefore, these trials were assigned as low or unclear regarding allocation concealment and sequence generation. Third, the SUCRA should be interpreted in agreement with the quality of evidence. In addition, as several outcomes have been investigated in the current study, the SUCRA should be considered specifically for each one. Moreover, the extent of differences in the effects between treatments is not considered during the computation of SUCRA and, equally important, SUCRA does not allow assessing any statistically significant difference. Last, in this study we pooled various dosage of one ASM into a single result, thus the difference in efficacy of multiple dosages of some ASMs have not been investigated.

## Conclusion

According to the SUCRA ranking, lamotrigine, levetiracetam, and oxcarbazepine were the three best ASMs. With regard to tolerability, oxcarbazepine was more likely to result in premature discontinuation and topiramate was associated with higher occurrence of side effects.

## Data Availability

The original contributions presented in the study are included in the article/Supplementary Material, further inquiries can be directed to the corresponding author.
