# Supplementary figures and images for: Comparative antiseizure medications of adjunctive treatment for children with drug-resistant focal-onset seizures: A systematic review and network meta-analysis
Source: Front Pharmacol. 2022 Dec 16;13:978876. doi: 10.3389/fphar.2022.978876 (PMC9800847; doi:10.3389/fphar.2022.978876)

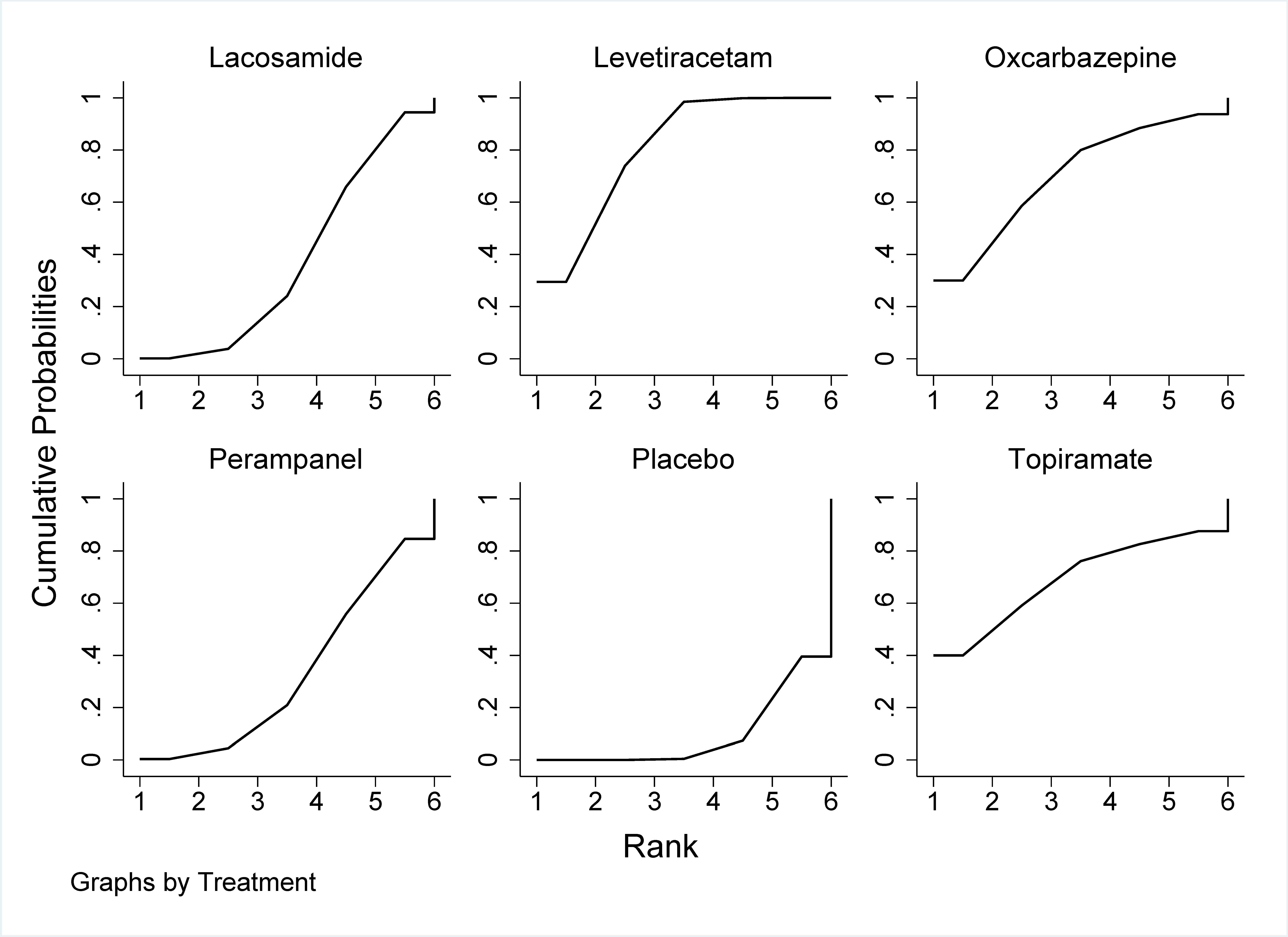

Supplement: Supplementary file 1 [file Image3.JPEG]

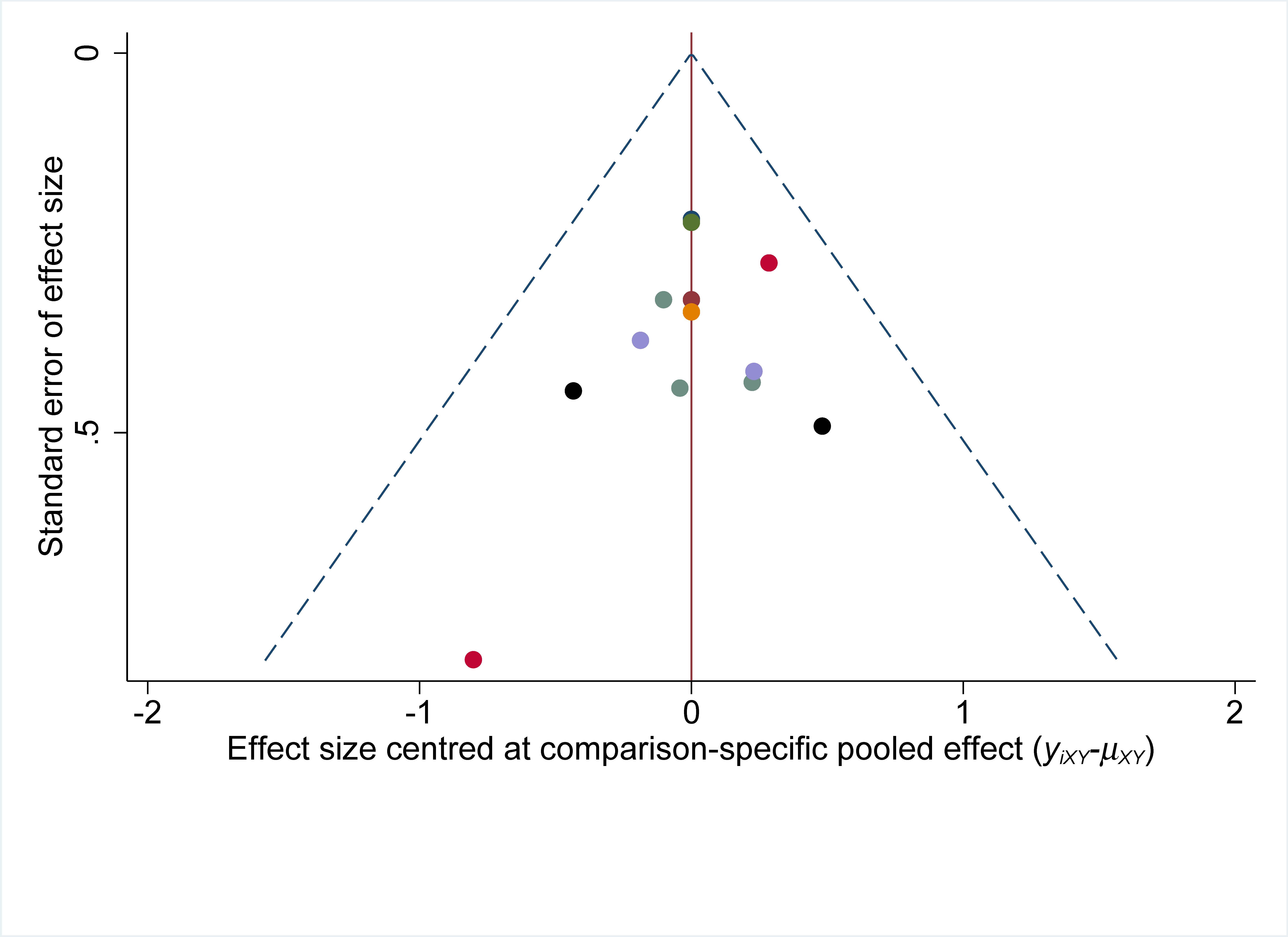

Supplement: Supplementary file 3 [file Image2.JPEG]
