# Supplementary material for: Comparative antiseizure medications of adjunctive treatment for children with drug-resistant focal-onset seizures: A systematic review and network meta-analysis
Source: Front Pharmacol. 2022 Dec 16;13:978876. doi: 10.3389/fphar.2022.978876 (PMC9800847; doi:10.3389/fphar.2022.978876)
Supplement: Supplementary file 4 [file DataSheet1.docx]

***Supplementary Table 1. Occurrence of Side Effect***

| **Lamotrigine** | 1.30 (0.57,2.96) | 1.28 (0.48,3.42) | 1.56 (0.58,4.20) | 1.98 (0.74,5.29) | 2.03 (0.73,5.60) | 2.58 (0.90,7.40) | 3.15 (1.13,8.74) | 5.34 (1.62,17.57) |
| --- | --- | --- | --- | --- | --- | --- | --- | --- |
| 0.77 (0.34,1.75) | **Placebo** | 0.99 (0.58,1.68) | 1.20 (0.69,2.08) | 1.52 (0.88,2.61) | 1.56 (0.86,2.83) | 1.99 (1.03,3.82) | 2.42 (1.32,4.43) | 4.11 (1.74,9.70) |
| 0.78 (0.29,2.08) | 1.01 (0.60,1.73) | **Eslicarbazepine** | 1.22 (0.57,2.61) | 1.54 (0.72,3.29) | 1.58 (0.71,3.51) | 2.01 (0.87,4.68) | 2.45 (1.09,5.49) | 4.16 (1.51,11.45) |
| 0.64 (0.24,1.73) | 0.83 (0.48,1.45) | 0.82 (0.38,1.77) | **Levetiracetam** | 1.27 (0.59,2.74) | 1.30 (0.58,2.93) | 1.66 (0.71,3.90) | 2.02 (0.89,4.59) | 3.43 (1.23,9.51) |
| 0.51 (0.19,1.36) | 0.66 (0.38,1.13) | 0.65 (0.30,1.39) | 0.79 (0.36,1.71) | **Lacosamide** | 1.03 (0.46,2.29) | 1.31 (0.56,3.06) | 1.59 (0.71,3.59) | 2.70 (0.98,7.47) |
| 0.49 (0.18,1.36) | 0.64 (0.35,1.16) | 0.63 (0.28,1.41) | 0.77 (0.34,1.73) | 0.97 (0.44,2.18) | **Perampanel** | 1.27 (0.53,3.09) | 1.55 (0.66,3.62) | 2.63 (0.93,7.50) |
| 0.39 (0.14,1.11) | **0.50 (0.26,0.97)** | 0.50 (0.21,1.15) | 0.60 (0.26,1.42) | 0.76 (0.33,1.79) | 0.78 (0.32,1.90) | **Gabapentin** | 1.22 (0.50,2.97) | 2.07 (0.70,6.09) |
| **0.32 (0.11,0.88)** | **0.41 (0.23,0.76)** | **0.41 (0.18,0.91)** | 0.50 (0.22,1.13) | 0.63 (0.28,1.42) | 0.64 (0.28,1.51) | 0.82 (0.34,2.00) | **Oxcarbazepine** | 1.70 (0.59,4.86) |
| **0.19 (0.06,0.62)** | **0.24 (0.10,0.58)** | **0.24 (0.09,0.66)** | **0.29 (0.11,0.81)** | 0.37 (0.13,1.02) | 0.38 (0.13,1.08) | 0.48 (0.16,1.43) | 0.59 (0.21,1.68) | **Topiramate** |

*Footnote: Network meta-analysis results of the efficacy (responder rate) in terms of odd ratios (ORs) and 95% confidence intervals. Bold values indicate* statistically significant difference (P<0.05)*.*

***Supplementary Table 2. Dropout for any reason***

| **Topiramate** | 1.55 (0.27,8.83) | 2.15 (0.42,11.10) | 2.16 (0.35,13.46) | 2.17 (0.33,14.19) | 2.69 (0.43,16.88) | 2.82 (0.74,10.79) | 3.52 (0.55,22.54) | 9.06 (1.43,57.38) |
| --- | --- | --- | --- | --- | --- | --- | --- | --- |
| 0.65 (0.11,3.68) | **Perampanel** | 1.39 (0.32,5.94) | 1.39 (0.26,7.37) | 1.40 (0.25,7.81) | 1.73 (0.32,9.25) | 1.82 (0.60,5.51) | 2.27 (0.42,12.38) | 5.84 (1.12,30.53) |
| 0.47 (0.09,2.40) | 0.72 (0.17,3.09) | **Levetiracetam** | 1.00 (0.21,4.79) | 1.01 (0.20,5.09) | 1.25 (0.26,6.01) | 1.31 (0.51,3.37) | 1.64 (0.33,8.06) | 4.21 (0.90,19.75) |
| 0.46 (0.07,2.89) | 0.72 (0.14,3.80) | 1.00 (0.21,4.75) | **Gabapentin** | 1.00 (0.16,6.15) | 1.24 (0.21,7.30) | 1.31 (0.38,4.54) | 1.63 (0.27,9.75) | 4.20 (0.73,24.18) |
| 0.46 (0.07,3.02) | 0.72 (0.13,3.99) | 0.99 (0.20,5.01) | 1.00 (0.16,6.10) | **Lamotrigine** | 1.24 (0.20,7.64) | 1.30 (0.35,4.85) | 1.63 (0.26,10.21) | 4.18 (0.69,25.35) |
| 0.37 (0.06,2.34) | 0.58 (0.11,3.08) | 0.80 (0.17,3.85) | 0.80 (0.14,4.71) | 0.81 (0.13,4.98) | **Lacosamide** | 1.05 (0.30,3.69) | 1.31 (0.22,7.90) | 3.37 (0.58,19.59) |
| 0.35 (0.09,1.36) | 0.55 (0.18,1.66) | 0.76 (0.30,1.96) | 0.77 (0.22,2.66) | 0.77 (0.21,2.87) | 0.95 (0.27,3.35) | **Placebo** | 1.25 (0.35,4.51) | 3.21 (0.94,11.01) |
| 0.28 (0.04,1.82) | 0.44 (0.08,2.40) | 0.61 (0.12,3.00) | 0.61 (0.10,3.66) | 0.62 (0.10,3.87) | 0.76 (0.13,4.59) | 0.80 (0.22,2.89) | **Eslicarbazepine** | 2.57 (0.43,15.22) |
| **0.11 (0.02,0.70)** | **0.17 (0.03,0.89)** | 0.24 (0.05,1.11) | 0.24 (0.04,1.37) | 0.24 (0.04,1.45) | 0.30 (0.05,1.72) | 0.31 (0.09,1.07) | 0.39 (0.07,2.30) | **Oxcarbazepine** |

*Footnote: Network meta-analysis results of the efficacy (responder rate) in terms of odd ratios (ORs) and 95% confidence intervals. Bold values indicate* statistically significant difference (P<0.05)*.*

***Supplementary Table 3. Dropout for side effect***

| **Perampanel** | 1.00 (0.07,14.22) | 1.07 (0.11,9.98) | 1.50 (0.20,11.32) | 1.31 (0.13,13.74) | 1.54 (0.26,9.02) | 3.41 (0.29,39.59) | 5.00 (0.46,53.99) | 7.36 (0.88,61.85) |
| --- | --- | --- | --- | --- | --- | --- | --- | --- |
| 1.00 (0.07,14.22) | **Topiramate** | 1.07 (0.10,11.89) | 1.50 (0.15,14.71) | 1.31 (0.11,16.24) | 1.54 (0.21,11.20) | 3.41 (0.25,46.48) | 5.00 (0.39,63.67) | 7.36 (0.69,78.24) |
| 0.94 (0.10,8.77) | 0.94 (0.08,10.45) | **Lacosamide** | 1.41 (0.24,8.33) | 1.23 (0.16,9.74) | 1.45 (0.37,5.71) | 3.20 (0.36,28.44) | 4.68 (0.57,38.43) | 6.90 (1.05,45.29) |
| 0.67 (0.09,5.04) | 0.67 (0.07,6.54) | 0.71 (0.12,4.22) | **Levetiracetam** | 0.88 (0.13,5.96) | 1.03 (0.33,3.20) | 2.28 (0.30,17.56) | 3.33 (0.47,23.59) | 4.91 (0.95,25.38) |
| 0.76 (0.07,7.96) | 0.76 (0.06,9.41) | 0.81 (0.10,6.43) | 1.14 (0.17,7.77) | **Lamotrigine** | 1.17 (0.25,5.52) | 2.60 (0.26,25.88) | 3.81 (0.41,35.12) | 5.60 (0.75,41.94) |
| 0.65 (0.11,3.79) | 0.65 (0.09,4.71) | 0.69 (0.18,2.73) | 0.97 (0.31,3.02) | 0.85 (0.18,4.00) | **Placebo** | 2.21 (0.40,12.11) | 3.24 (0.66,15.97) | 4.77 (1.32,17.28) |
| 0.29 (0.03,3.40) | 0.29 (0.02,3.99) | 0.31 (0.04,2.78) | 0.44 (0.06,3.39) | 0.38 (0.04,3.83) | 0.45 (0.08,2.47) | **Gabapentin** | 1.46 (0.14,15.07) | 2.16 (0.26,18.18) |
| 0.20 (0.02,2.16) | 0.20 (0.02,2.55) | 0.21 (0.03,1.75) | 0.30 (0.04,2.12) | 0.26 (0.03,2.43) | 0.31 (0.06,1.52) | 0.68 (0.07,7.03) | **Eslicarbazepine** | 1.47 (0.19,11.44) |
| 0.14 (0.02,1.14) | 0.14 (0.01,1.44) | **0.14 (0.02,0.95)** | 0.20 (0.04,1.05) | 0.18 (0.02,1.34) | **0.21 (0.06,0.76)** | 0.46 (0.05,3.91) | 0.68 (0.09,5.28) | **Oxcarbazepine** |

*Footnote:* *Network meta-analysis results of the efficacy (responder rate) in terms of odd ratios (ORs) and 95% confidence intervals. Bold values indicate* statistically significant difference (P<0.05)*.*

***Supplementary Table 4. The safety and tolerability profile***

| **Study** | **Treatment** | **Any**  **Adverse Event**  **(Active/PBO)** | **Dropouts**  **for Any Reason**  **(Active/PBO)** | **Dropouts for Adverse Event**  **(Active/PBO)** | **Serious**  **Adverse Events**  **(Active/PBO)** | **Most Common**  **Adverse Events (≥5%)**  **(Active Treatment)** |
| --- | --- | --- | --- | --- | --- | --- |
| Lagae | Perampanel | 68/31 | 9/5 | 3/0 | 5/2 | Dizziness, Somnolence, Headache, Fatigue, Aggression, Irritability, Weight increased, Convulsion, Nasopharyngitis, URTI, Insomnia |
| Glauser | Levetiracetam | 89/89 | 7/14 | 5/9 | 8/9 | Somnolence, Accidental injury, Vomiting, Anorexia, Rhinitis, Hostility, Cough, Pharyngitis, Nervousness, Asthenia, Diarrhea, Personality disorder, Dizziness, Emotional lability, Pain, Agitation |
| Duchowny | Lamotrigine | 92/96 | 14/18 | 5/6 | 2/0 | Vomiting, Somnolence, Infection, Dizziness, Rash, Headache, Rhinitis, Accidental injury, Diarrhea, Fever, Abdominal pain, Tremor, Nausea, Otitis media, Pharyngitis, Ataxia, Asthenia |
| Elterman | Topiramate | NA | 0/2 | 0/1 | 3/1 | URTI, Sinusitis, Coughing, Diarrhea, Somnolence, Mood problems, Aggressive reaction, Nervousness, Rash, Otitis media, Fever, Injury, Fatigue |
| Glauser | Oxcarbazepine | 124/106 | 21/10 | 14/4 | 2/2 | Somnolence, Headache, Dizziness, Ataxia, Abnormal gait, Nystagmus, Vomiting, Nausea, Abdominal pain, Anorexia, Fever, Fatigue, Rhinitis, Pharyngitis, URT, Diplopia, Abnormal vision, Infections |
| Piña-Garza | Levetiracetam | 33/25 | 2/3 | 2/1 | 1/1 | Pyrexia, Somnolence, Irritability |
| Farkas | Lacosamide | 116/100 | 20/21 | 7/10 | 8/10 | Somnolence, Dizziness, Nasopharyngitis, Vomiting, Pyrexia, Headache, URTI |
| Appleton | Gabapentin | 40/26 | 21/28 | 6/3 | 9/3 | Viral infection, Fever, Nausea, vomiting, Somnolence, Pharyngitis, Hostility, URTI, Headache, Rhinitis |
| Rosenfeld | Perampanel | 70/31 | 6/8 | 2/3 | 3/3 | Dizziness, Somnolence, Nasopharyngitis, Aggression, Headache, Convulsion, Pyrexia |
| Novotny | Topiramate | 91/19 | 11/8 | 4/2 | 9/3 | Fever, Ataxia, Diarrhea, URTI Cough Somnolence Vomiting |
| Levisohn | Levetiracetam | 57/29 | 14/5 | 7/2 | 5/2 | Headache, URTI, Abdominal pain, Nasopharyngitis, Fatigue, Vomiting, Somnolence, Aggression, Dizziness, Nasal congestion, Pyrexia, decreased appetite, Abnormal behavior, Psychomotor hyperactivity, Cough, Insomnia, Altered mood, Anxiety |
| NCT00975715 | Oxcarbazepine | 38/27 | 9/1 | 8/1 | 1/1 | Constipation, Vomiting, Nasopharyngitis, URTI, Somnolence, Rash |
| NCT01527513 | Eslicarbazepine Acetate | 35/21 | 8/3 | 1/3 | 3/2 | Headache, Somnolence, Dizziness, Ataxia, Diplopia, Nausea, Vomiting, Fatigue, Anxiety, Insomnia, Irritability |
| NCT00988156 | Eslicarbazepine Acetate | 112/94 | 20/17 | 5/0 | 15/9 | Headache, Somnolence, Dizziness, Ataxia, Diplopia, Nausea, Vomiting, Fatigue, Anxiety, Insomnia, Irritability |

**Abbreviations**: NA, not available; PBO, placebo; URTI, upper respiratory tract infection.
